# Modified Gait Support in Adults Three to Eighteen Months After Concussion

**DOI:** 10.3390/s26144346

**Published:** 2026-07-09

**Authors:** Tyler A. Wood, Nicholas E. Grahovec

**Affiliations:** College of Health and Human Sciences, School of Rehabilitation and Performance Sciences, Northern Illinois University, DeKalb, IL 60115, USA; ngrahovec@niu.edu

**Keywords:** clinical testing, health monitoring, motor control, traumatic brain injury

## Abstract

**Highlights:**

**What are the main findings?**

- Individuals with prior concussion demonstrated shorter pressure-walkway-derived step length during obstacle walking, with altered support-phase timing patterns across task conditions.
- Group differences were more pronounced under obstacle conditions, suggesting persistent deficits in sensorimotor control and movement planning months after concussion.

**What are the implications of the main findings?**

- Standard clinical measures, including time to completion, gait velocity, and cognitive-task accuracy, did not differentiate groups, highlighting the added value of pressure-walkway-derived gait metrics.
- More research is needed to identify systems to monitor how these motor control deficits contribute to subsequent risk of musculoskeletal injury.

**Abstract:**

Concussion is associated with persistent motor control deficits that may not be detected using standard clinical assessments. This study examined differences in average velocity, average step length, and single- and double-support percentages during gait under increasing task demands in individuals with a history of concussion. Sixty participants aged 18 to 35 years were recruited, including 32 individuals with a concussion within the past 3 to 18 months and 28 healthy controls. Gait data were collected using an instrumented pressure-sensitive walkway across four conditions: single-task and dual-task walking, with and without obstacles. Repeated-measures analyses of covariance were used to assess group and condition effects, with sex as a covariate, which showed a significant group-by-condition effect for step length, single-support percentage, and double-support percentage. These findings identify step length, single-support percentage, and double-support percentage as candidate sensor-derived gait biomarkers for detecting persistent post-concussion motor control alterations. The results directly support the use of pressure-based gait sensing to quantify deficits missed by conventional clinical measures; however, future work is needed to determine whether these features translate to wearable or real-world monitoring systems.

## 1. Introduction

Concussion remains a common injury in physically active populations and presents persistent challenges for clinicians who monitor recovery and readiness to return to activity [1]. Traditional clinical assessments rely heavily on static balance tasks and tandem gait to evaluate postural control [2,3,4]. These approaches offer practical value and are widely implemented [2,3]. Despite their utility, these measures capture performance under constrained and low-demand conditions, which may not reflect the complexity of real-world movement [4,5].

Emerging research highlights that concussion disrupts central motor control processes, including sensory integration, neuromuscular coordination, and anticipatory movement planning [5,6]. These disruptions affect dynamic tasks such as walking, especially when attention is divided or when environmental demands increase [5,6]. Gait requires continuous adjustments to internal and external inputs, and impairments in this system may persist despite normal findings on static assessments [5,6]. Studies using instrumented gait analysis have identified altered stride variability, reduced gait speed, and changes in temporal–spatial parameters months after concussion [7,8]. These findings suggest that current clinical tools may lack the sensitivity to detect lingering deficits in motor control [4,5].

A growing concern involves the association between prior concussion and increased risk of subsequent musculoskeletal injury [4]. Individuals with a history of concussion demonstrate higher rates of lower-extremity musculoskeletal injury following return to activity [4,6,9]. This elevated risk is thought to stem from unresolved neuromuscular and motor control impairments that affect movement quality during functional tasks [4,6,9]. As a result, there is a need to better understand how concussion affects motor planning and execution during functional activities.

Dual-task paradigms and obstacle negotiation have emerged as more sensitive approaches for assessing motor control following concussion [10,11]. These tasks introduce cognitive and environmental demands that challenge attentional resources and reveal deficits not observed under single-task conditions [4]. Dual-task gait assessments have shown that individuals with a history of concussion exhibit greater performance decrements when cognitive load is added, indicating impaired attentional allocation and motor control integration [12]. Similarly, obstacle-crossing tasks require precise coordination and anticipatory adjustments, which may reveal deficits in movement planning [11].

One area that remains underexplored is how concussions influence the distribution of support phases during gait in adults. Single- and double-leg support percentages reflect the temporal organization of gait and provide insight into stability and motor control strategies [13]. Changes in these parameters may indicate compensatory mechanisms or altered neuromuscular control [13]. Single- and double-support percentages were selected because they represent biomechanically meaningful phases of gait. Single support requires the individual to maintain the body’s center of mass over one limb, controlling forward progression and preparing for the next step. This phase depends on lower-extremity strength, sensorimotor integration, anticipatory postural control, and dynamic balance [13]. In contrast, double support occurs when both feet are in contact with the ground and reflects the transition between limbs during weight transfer. Therefore, concussion-related reductions in single support and increases in double support may indicate altered dynamic stability, reduced confidence in unilateral loading, or compensatory movement strategies during higher-demand walking tasks [5]. Examining these variables across varying task demands may provide a more detailed understanding of how individuals adapt their movement following concussion [5]. However, these variables cannot be captured through standard clinical testing. Thus, there is a need for more ecologically valid assessments that capture the complexity of real-world movement.

Pressure-sensitive gait sensing provides a practical method for quantifying foot-contact timing and spatial footfall patterns during higher-demand walking tasks [14,15]. This study contributes to sensor-based concussion assessment by identifying which walkway-derived spatiotemporal features are sensitive to persistent gait alterations after concussion. Rather than relying on visible clinical performance, total task time, or standard gait speed alone, the pressure-sensitive walkway quantified spaciotemporal measures across increasing task demands [15]. These features provide objective indicators of gait organization during obstacle negotiation and represent candidate sensor-derived gait biomarkers for post-concussion assessment.

Therefore, the purpose of this study was to examine pressure-walkway-derived gait features during cognitive and obstacle walking conditions in individuals who sustained a concussion three to 18 months prior to testing, compared with healthy controls. Specifically, this study examined average velocity, step length, single-support percentage, and double-support percentage as objective indicators of gait organization. This work aims to identify sensor-derived features associated with persistent post-concussion motor control alterations and to clarify which measures warrant future validation in wearable or real-world monitoring systems [15,16].

## 2. Materials and Methods

### 2.1. Study Design and Participants

This cross-sectional study was conducted in a controlled university laboratory setting. Sixty participants, aged 18 to 35 years, were recruited and divided into two groups. The concussion group included 32 individuals with a history of concussion within the past 3 to 18 months and were medically cleared to return to activity. The control group included 28 healthy individuals with no previous concussion history. Exclusion criteria included concussion within 3 months, current post-concussion syndrome symptoms, lower-extremity injury, vestibular or neurological disorders, or use of medications affecting balance. All study procedures were approved by the University’s Institutional Review Board, and participants provided informed consent prior to data collection.

### 2.2. Instrumentation

Demographic and clinical information was collected, including age, sex, height, weight (for body mass index [BMI]), number of lower-extremity musculoskeletal injuries in the previous year number of prior concussions, time since concussion, the number of weeks of recovery, if there were any symptoms after medical clearance for the concussion group, and Montreal Cognitive Assessment (MoCA) [17,18]. Gait data were collected using the Zeno Pressure Sensitive Walkway (ProtoKinetics LLC, Havertown, PA, USA). This instrumented walkway consists of an array of embedded capacitive pressure sensors with a spatial resolution of approximately 1.27 cm and a sampling frequency of 120 Hz. The system detects foot contact events based on pressure thresholds applied across adjacent sensors. Temporal event detection, including initial contact and toe-off, is derived using proprietary algorithms within the PKMAS Version 503c9 software (ProtoKinetics LLC, Havertown, PA, USA). Reported measurement error for temporal parameters is less than 1 percent, with high test–retest reliability for spatiotemporal gait metrics [14]. Raw sensor data are processed through a multi-step pipeline that includes signal filtering, footfall segmentation, and gait cycle normalization. Each foot contact is mapped to a sequence of activated sensors. The active sensing area measured 3.7 m and was integrated within a 4.5 m walking course to allow for steady-state gait. The PKMAS system software processed footfall data and calculated the average velocity, average step length, and the percentage of time spent in single- and double-support phases. Output variables are averaged across trials to reduce variability and improve measurement stability.

### 2.3. Experimental Procedures

**Figure 1:**
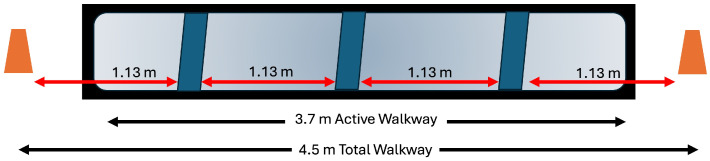
Walkway illustration with distances between obstacles.

Participants completed four walking conditions in a randomized order using a random number generator. These included single-task walking without obstacles, dual-task walking without obstacles, single-task walking with obstacles, and dual-task walking with obstacles. Each participant performed three trials per condition, and the time to complete each trial was recorded. Participants were instructed to walk at a normal, comfortable pace with athletic shoes. No practice trials were given. For dual-task conditions, participants completed the serial 7 s cognitive task while walking. This task involved serially subtracting sevens from a randomly assigned three-digit number. For the obstacle course, three hurdles, each 20 cm high, were placed at equal intervals (1.3 m) along the walkway (Figure 1). During the dual-task trials, the number of utterances and the number of correct utterances were collected. Any trials that resulted in errors (i.e., walking off the mat and incomplete data recording) were repeated. These conditions were designed to increase cognitive and mechanical demands during gait.

### 2.4. Outcome Measures

The primary outcome measures were velocity, step length, and the percentages of time spent in single- and double-leg support per gait cycle, as measured by the PKMAS Software. The system software calculated these values for each trial based on the pressure sensor input. The average of three trials per condition was used for analysis.

### 2.5. Statistical Analysis

Age, BMI, and MoCA scores were summarized as mean ± SD and compared between groups using independent-samples *t*-tests. Sex and lower-extremity musculoskeletal injury history in the previous year were summarized with frequency counts and compared between groups using chi-square tests. For the concussion group, number of previous concussions, time since concussion, weeks to recovery, and residual symptoms after medical clearance were summarized descriptively. Dual-task cognitive performance was summarized by the number of utterances, number of correct responses, and percentage of correct responses. Percentage of correct responses was analyzed separately for the no-obstacle and obstacle dual-task conditions using analysis of covariance (ANCOVA), with group as the between-subject factor and sex as a covariate.

Time to completion, average gait velocity, average step length, single-support percentage, and double-support percentage were analyzed using separate repeated-measures analyses of covariance (RMANCOVAs). Condition served as the within-subject factor with four levels: single task with no obstacle, dual task with no obstacle, single task with obstacle, and dual task with obstacle. Group served as the between-subject factor. Sex was included as a covariate due to known sex differences after concussion [19]. Type III sums of squares were used. Mauchly’s test assessed sphericity. Sphericity-assumed values were reported when this assumption was met, and Greenhouse–Geisser-corrected values were reported when sphericity was violated. For each model, the main effect of condition, main effect of group, and group-by-condition interaction were reported. Estimated marginal means, 95% confidence intervals, adjusted mean differences, and Bonferroni-adjusted pairwise comparisons were used to interpret significant main effects or interactions. Effect sizes were reported as partial eta squared (ηp^2^) and converted to Cohen’s f. Cohen’s f values were interpreted as small (<0.25), medium (0.25 to <0.40), or large (≥0.40) [20]. Statistical significance was set a priori at *p* < 0.05. Analyses were conducted using SPSS Version 29 (IBM Corp., Armonk, NY, USA). Analyses used all valid complete cases for each outcome model, so the analytic sample size varied by outcome. A posteriori power analysis was conducted in G*Power Version 3.1 (HHU, Düsseldorf, Germany) for the primary support-phase outcomes using the observed effect sizes, analytic sample size, and α = 0.05.

### 2.6. Generative AI Statement

The authors used generative artificial intelligence tools (ChatGPT Version 5.3) to support the preparation of this manuscript. These tools assisted with language editing, grammar refinement, and text organization. The authors reviewed and revised all AI-generated content to ensure accuracy, clarity, and alignment with the study’s methods and findings. No generative AI tools were used for data collection, data analysis, or interpretation of results. All scientific decisions, analyses, and conclusions were made solely by the authors. The authors take full responsibility for the integrity and originality of the work.

## 3. Results

### 3.1. Participants

Thirty-two participants (18 males/14 females; age 20.9 ± 1.7 years) who sustained a previous concussion and 28 health controls (12 males/16 females; age 21.5 ± 1.7 years) completed all testing procedures. There was no significant difference in age between the groups (*p* = 0.283). The concussion group reported sustaining 2.2 ± 1.8 concussions, with the most recent concussion being reported 6.5 ± 0.6 months prior to testing, and it took an average of 3.3 ± 2.7 weeks to recover. Within the concussion group, 50% (*n* = 16) reported experiencing residual concussion symptoms even after being medically cleared. Body mass index of the concussed group was 25.6 ± 4.5, compared to 25.9 ± 3.7 of the control, with no significant group difference (*p* = 0.438). Montreal Cognitive Assessment Score of the concussed group was 24.8 ± 3.3, compared to 26.6 ± 2.1 of the healthy controls, with no significant group difference (*p* = 0.100). When asked about the number of lower-extremity injuries in the previous year, three members of the concussion group and seven members of the control group reported experiencing an injury. Chi-square analysis revealed no significant group differences, χ(1) = 2.625, *p* = 0.105.

### 3.2. Gait Data

Table 1 presents the mean trial time for each condition. For time to completion, Mauchly’s test indicated that the assumption of sphericity was violated, W = 0.704, χ^2^(5) = 19.237, and *p* = 0.002. Greenhouse–Geisser-corrected values are reported. The repeated-measures ANCOVA revealed no significant main effect of condition: F(2.513, 140.751) = 1.420, *p* = 0.243, ηp^2^ = 0.025, and f = 0.16. The main effect of group was not significant: F(1, 56) = 0.766, *p* = 0.385, ηp^2^ = 0.013, and f = 0.11. The group-by-condition interaction was also not significant: F(2.513, 140.751) = 0.055, *p* = 0.970, ηp^2^ = 0.001, and f = 0.03. Simple group comparisons showed no significant differences between groups in any condition. The adjusted mean difference was 0.63 s, 95% CI [−0.68, 1.94], and *p* = 0.340 during single-task walking without obstacles; 0.72 s, 95% CI [−1.20, 2.64], and *p* = 0.455 during dual-task walking without obstacles; 0.53 s, 95% CI [−1.35, 2.40], and *p* = 0.577 during single-task obstacle walking; and 0.81 s, 95% CI [−1.16, 2.79], and *p* = 0.412 during dual-task obstacle walking. These results indicate that time to completion did not differentiate the concussion and control groups.

Table 2 presents the average number of utterances, the number of correct responses, and the percentage of correct responses during the dual task trials. For the no-obstacle conditions, there was no significant group difference (F(1, 54) = 3.869; *p* = 0.054) in the percentage of correct responses, with a medium effect size (f = 0.27). For the obstacle conditions, there was no significant group difference (F(1, 55) = 1.454; *p* = 0.233) in the percentage of correct responses, with a small effect size (f = 0.16).

Table 3 presents the average gait velocity and step length across walking conditions for each group. For gait velocity, Mauchly’s test indicated that the assumption of sphericity was not violated: W = 0.862, χ^2^(5) = 7.398, and *p* = 0.193. Sphericity-assumed values are reported. The repeated-measures ANCOVA revealed no significant main effect of condition: F(3, 153) = 1.390, *p* = 0.248, ηp^2^ = 0.027, and f = 0.17. The main effect of group was not significant: F(1, 51) = 1.278, *p* = 0.264, ηp^2^ = 0.024, and f = 0.16. The group-by-condition interaction was also not significant: F(3, 153) = 0.413, *p* = 0.744, ηp^2^ = 0.008, and f = 0.09. The adjusted mean gait velocity across conditions was 98.75 cm/s for the concussion group, 95% CI [93.04, 104.46], and 103.43 cm/s for the control group, 95% CI [97.50, 109.36]. The adjusted mean difference between groups was −4.68 cm/s, 95% CI [−12.98, 3.63], and *p* = 0.264. Simple group comparisons showed no significant group differences in any walking condition: single task with no obstacle, adjusted mean difference = −5.12 cm/s, 95% CI [−13.23, 2.99], and *p* = 0.211; dual task with no obstacle, adjusted mean difference = −2.13 cm/s, 95% CI [−11.80, 7.54], and *p* = 0.660; single task with obstacle, adjusted mean difference = −5.60 cm/s, 95% CI [−16.26, 5.07], and *p* = 0.297; and dual task with obstacle, adjusted mean difference = −5.85 cm/s, 95% CI [−15.32, 3.61], and *p* = 0.220.

For step length, Mauchly’s test indicated that the assumption of sphericity was violated: W = 0.274, χ^2^(5) = 64.431, and *p* < 0.001. Greenhouse–Geisser-corrected values are reported. The repeated-measures ANCOVA revealed a significant main effect of condition: F(2.035, 103.772) = 6.281, *p* = 0.003, ηp^2^ = 0.110, and f = 0.35. The main effect of group was significant: F(1, 51) = 7.356, *p* = 0.009, ηp^2^ = 0.126, and f = 0.38. There was also a significant group-by-condition interaction: F(2.035, 103.772) = 3.814, *p* = 0.025, ηp^2^ = 0.070, and f = 0.27. Bonferroni-adjusted pairwise comparisons showed that the concussion group had significantly shorter step length than controls during single-task walking without obstacles, adjusted mean difference = −3.30 cm, 95% CI [−6.58, −0.02], and *p* = 0.049; single-task obstacle walking, adjusted mean difference = −8.83 cm, 95% CI [−16.76, −0.89], and *p* = 0.030; and dual-task obstacle walking, adjusted mean difference = −10.68 cm, 95% CI [−17.90, −3.45], and *p* = 0.005. The group difference during dual-task walking without obstacles was not significant: adjusted mean difference = −2.41 cm, 95% CI [−6.09, 1.27], and *p* = 0.194.

Table 4 presents the percentages of single- and double-support for each walking condition and group. For single-support percentage, Mauchly’s test indicated that the assumption of sphericity was violated: W = 0.706, χ^2^(5) = 17.321, and *p* = 0.004. Greenhouse–Geisser-corrected values are reported. The repeated-measures ANCOVA revealed a significant main effect of condition: F(2.543, 129.701) = 56.410, *p* < 0.001, ηp^2^ = 0.525, and f = 1.05. The main effect of group was not significant: F(1, 51) = 1.058, *p* = 0.309, ηp^2^ = 0.020, and f = 0.14. There was a significant group-by-condition interaction: F(2.543, 129.701) = 4.099, *p* = 0.012, ηp^2^ = 0.074, and f = 0.28. The group-by-condition interaction reflected larger group differences during obstacle walking than during no-obstacle walking. Simple group comparisons showed no significant group differences during single-task walking without obstacles, adjusted mean difference = 0.06%, 95% CI [−0.82, 0.94], and *p* = 0.889; or dual-task walking without obstacles, adjusted mean difference = −0.06%, 95% CI [−1.02, 0.89], and *p* = 0.893. During obstacle conditions, the concussion group demonstrated lower adjusted single-support percentages than controls; however, these simple group differences did not reach statistical significance. The adjusted mean difference was −0.97%, 95% CI [−2.01, 0.07], and *p* = 0.067 during single-task obstacle walking, and −0.76%, 95% CI [−1.67, 0.16], and *p* = 0.102, during dual-task obstacle walking.

For double-support percentage, Mauchly’s test indicated that the assumption of sphericity was violated: W = 0.732, χ^2^(5) = 15.482, and *p* = 0.009. Greenhouse–Geisser-corrected values are reported. The repeated-measures ANCOVA revealed a significant main effect of condition: F(2.476, 126.258) = 66.558, *p* < 0.001, ηp^2^ = 0.566, and f = 1.14. The main effect of group was not significant: F(1, 51) = 0.681, *p* = 0.413, ηp^2^ = 0.013, f = 0.11. There was a significant group-by-condition interaction: F(2.476, 126.258) = 4.139, *p* = 0.012, ηp^2^ = 0.075, and f = 0.28. The group-by-condition interaction reflected larger group differences during obstacle walking than during no-obstacle walking. Simple group comparisons showed no significant group differences during single-task walking without obstacles, adjusted mean difference = −0.09%, 95% CI [−1.84, 1.66], and *p* = 0.918, or dual-task walking without obstacles, adjusted mean difference = −0.17%, 95% CI [−2.08, 1.73], and *p* = 0.856. During obstacle conditions, the concussion group demonstrated higher adjusted double-support percentages than controls; however, these simple group differences did not reach statistical significance. The adjusted mean difference was 1.49%, 95% CI [−0.40, 3.39], and *p* = 0.120 during single-task obstacle walking, and 1.47%, 95% CI [−0.28, 3.21], and *p* = 0.098 during dual-task obstacle walking.

A posteriori power analysis was conducted using the percentage of time in single- and double-support, both of which had a medium effect (f = 0.28). Using α = 0.05, the analysis indicated that this study had a power of 36.9%.

## 4. Discussion

The purpose of this study was to examine pressure-walkway-derived gait features during increasingly demanding walking conditions in young adults with a history of concussion. The primary finding was that group differences emerged, particularly under obstacle conditions. The concussed group demonstrated shorter step length, reduced single-support percentage, and greater double-support percentage than controls during obstacle conditions, although pairwise comparisons did not show a significant adjusted difference for single- and double-support percentages. In healthy gait, single-support typically accounts for about 38 to 40 percent of the gait cycle per limb, while double-support accounts for about 20 to 24 percent, reflecting efficient forward progression and dynamic stability [21]. While both the healthy controls and concussed participants displayed values consistent with these standards, the decreased step-length findings with the single- and double-support percentages may reflect a shift toward a more conservative movement strategy that prioritizes stability as environmental demands increase. These findings indicate that motor control alterations persist beyond clinical recovery and become evident only when tasks require higher levels of coordination and adaptability, aligning with evidence of lingering sensorimotor deficits after concussion despite apparent recovery in young adults [4]. The primary sensing contribution of this study is the identification of pressure-walkway-derived gait features that differentiated individuals with a history of concussion from healthy controls during higher-demand walking. Potential clinical measures such as total trial time, gait velocity, and cognitive task accuracy did not clearly differentiate between groups. In contrast, step length, with nonsignificant supporting changes in single-support and double-support percentages, revealed group differences during obstacle walking. This pattern suggests that sensor-derived footfall and support-phase metrics provide additional measurement value beyond visible clinical performance and global walking outcomes.

The support-phase findings should be interpreted alongside the step-length results and as between-group differences within the same walking condition. Table 4 shows that both groups increased single-support and decreased double-support during obstacle walking relative to no-obstacle walking. Within the obstacle conditions, the concussion group showed shorter step length, lower single-support, and higher double-support than controls. Thus, the support-phase results reflect a relative between-group difference during obstacle negotiation, not an absolute increase in double-support caused by obstacles. These patterns suggest a relatively more conservative support strategy during obstacle negotiation, but the simple group comparisons for support-phase percentages did not reach statistical significance [4,5].

Taken together, the shorter step lengths and the pattern of a relatively decreased percentage of time in single-support and a relatively increased percentage of time in double-support compared to healthy controls suggest that the concussion group may have reduced forward progression and a relatively higher percentage of double-support than controls within obstacle conditions, despite both groups showing lower double-support during obstacle walking than during no-obstacle walking [4,5]. This pattern is consistent with a cautious gait strategy rather than an isolated measurement artifact [4,5]. Although the relative support-phase differences occurred alongside shorter step length during obstacle walking, their clinical significance remains uncertain. Exceeding reported measurement error does not establish that these differences exceed a minimal detectable change or clinically important difference. In addition, the reported <1% measurement error for temporal gait parameters may not directly apply to support-phase percentages during dual-task or obstacle walking. Therefore, these findings should be interpreted as preliminary sensor-derived group differences rather than established clinically meaningful changes.

The magnitude and direction of the present findings are consistent with prior gait studies after concussion. Previous work has shown that individuals with concussion or concussion history often demonstrate a more conservative gait strategy, including slower gait velocity, shorter stride or step length, reduced single-limb support, increased double-limb support, and altered center-of-mass control [5,7,8,11]. The present findings extend this work by showing that similar alterations are still evident three to 18 months after concussion, particularly when participants negotiated obstacles.

Obstacle walking appeared more sensitive than dual-task walking alone because it imposed a direct mechanical and visuospatial demand on gait. Dual-task walking increased cognitive load, but it did not require participants to alter foot placement, step length, limb trajectory, or anticipatory postural control to the same extent as obstacle negotiation. This may explain why group differences were minimal during no-obstacle walking but became more apparent during obstacle conditions. The dual-task obstacle condition did not yield a larger effect than the single-task obstacle condition, despite being the most demanding. One possible explanation is that the obstacle itself was the primary driver of the gait adaptation, while the added cognitive task produced similar slowing or prioritization strategies in both groups. Therefore, the findings suggest that mechanical and visuospatial demands may be more sensitive than cognitive load alone for identifying persistent gait alterations after concussion in this sample.

Prior work shows that individuals with concussion-related motor control deficits demonstrate altered biomechanics during functional tasks, which are linked to injury-related movement patterns [4,22]. The present results suggest that persistent post-concussion motor control deficits may remain clinically relevant after return to activity. Sport performance depends on rapid transitions through single-limb support during cutting, landing, and acceleration. The relatively reduced single-support time suggests decreased tolerance for unilateral loading, which may impair force production and movement efficiency [23]. The relatively higher double-support percentage in the concussion group during obstacle conditions may reflect greater reliance on bilateral contact than in controls [24].

The absence of meaningful group differences during no-obstacle walking reinforces the limitations of traditional clinical assessments. Standard return-to-play tools often rely on straight-line gait or static balance, which fails to replicate functional movement demands [4,25,26,27]. In contrast, obstacle negotiation requires anticipatory control, coordination, and precise foot placement. These findings support the need for more ecologically valid assessments; the obstacle paradigms tested here may better reflect the environments individuals encounter and are more likely to reveal persistent deficits that affect performance and safety [7,28].

A growing body of evidence shows that individuals with a history of concussion have a significantly elevated risk of subsequent musculoskeletal injury. Herman and colleagues reported that individuals were about 1.6 times more likely to sustain a lower-extremity injury following concussion [29]. Paterno and colleagues demonstrated that altered neuromuscular control contributes to abnormal joint loading and increased injury risk [30]. Fino and colleagues showed that subtle changes in gait timing, including support phases, reflect impaired dynamic stability [31]. Together, these findings may support the clinical relevance of the spaciotemporal changes observed in this study and may suggest that even small alterations in gait may contribute to elevated injury risk after medical clearance.

Several limitations should be considered. The sample consisted of young, physically active individuals, which may limit generalizability. Although these findings identify step length, single-support percentage, and double-support percentage as candidate sensor-derived gait variables, the present study does not establish their diagnostic accuracy, classification performance, or monitoring validity. The modest sample size and cross-sectional design limit the ability to develop stable prediction models or identify clinically meaningful thresholds. Variability in time since concussion may have influenced the magnitude of observed deficits. This study focused on spatiotemporal gait parameters and did not include joint kinematics and muscle activation, both of which are important for understanding the underlying mechanisms. These outcomes do not provide the same mechanistic information as kinematic or kinetic analyses. Step length and support-phase timing identify how participants organized gait during task performance, but they do not determine the joint-level movement patterns, loading strategies, or muscular contributions underlying these changes. The laboratory-based walkway limits insight into movement behavior in real-world environments. Additionally, we collected only self-reported vestibular or neurological problems. Since we did not physically screen the participants, there may be unmeasured visual, vestibular, or neurological factors that may confound the findings.

Future research should use longitudinal designs to examine how changes in support phases evolve over time and relate to injury outcomes in individuals. Combining spatiotemporal measures with biomechanical and neuromuscular data may improve understanding of how motor control deficits contribute to injury risk. Future studies should incorporate three-dimensional kinematics, ground reaction forces, joint moments, and neuromuscular measures to determine whether the observed spatial and temporal alterations reflect changes in joint excursion, limb loading, dynamic stability, or compensatory muscle activation after concussion. Intervention studies should evaluate whether targeted neuromuscular and dual-task training can restore normal support phase distribution and reduce injury incidence.

Future work may use these laboratory findings to guide the development of wearable or real-world monitoring approaches. The spatial and temporal variables identified in this study, including step length, single-support, and double-support, are candidate metrics that could be examined using wearable inertial sensors or instrumented insoles. These technologies may allow clinicians to assess gait during daily activity, training, or sport-specific movement. However, future studies must first determine whether these same gait alterations are detectable outside the laboratory and whether they relate to symptoms, recovery status, or subsequent musculoskeletal injury risk [14,32]. From an applied perspective, these findings support the development of sensor-based monitoring systems that target temporal gait features associated with post-concussion motor control. The relative reductions in single-support and relative increases in double-support observed in the concussion group during obstacle conditions provide candidate targets for future wearable detection algorithms. Inertial measurement units placed on the foot, shank, or pelvis can estimate stance and swing phases using acceleration and angular velocity signals [32]. Pressure-sensitive insoles provide direct measurement of ground-contact timing and can quantify support phase distribution outside the laboratory [32]. These systems enable the detection of small measurable differences in gait timing that are not observable through standard clinical assessment. Integration of these metrics into real-time feedback systems may improve clinical decision-making and rehabilitation targeting.

These findings identify step length, single-support percentage, and double-support percentage as candidate sensor-derived gait biomarkers for post-concussion assessment. This interpretation should remain cautious. The present study does not establish diagnostic accuracy, clinical thresholds, or monitoring validity. Rather, the findings identify measurable gait features that appear sensitive to persistent post-concussion motor control alterations under higher-demand walking conditions. Pressure-sensitive walkways offer controlled measurement of foot-contact timing and spatial footfall patterns, while wearable inertial sensors and instrumented insoles may eventually allow similar gait features to be assessed outside the laboratory. Future work should test whether single-support percentage, double-support percentage, and step length remain reliable and valid during daily activity, sport-specific movement, or rehabilitation tasks. Future studies should determine whether these same features predict recovery status, subsequent musculoskeletal injury, or response to rehabilitation.

## 5. Conclusions

Young adults with a history of concussion demonstrated a task-specific gait pattern during obstacle negotiation, characterized by shorter step length and nonsignificant support-phase differences toward lower single-support and higher double-support compared with controls. Both groups showed lower double-support during obstacle walking than during no-obstacle walking, the support-phase interpretation reflects between-group differences within obstacle conditions rather than an obstacle-induced increase in double-support. Although the small support-phase differences warrant cautious interpretation, the combined spatial and temporal changes may suggest a more conservative gait strategy under higher-demand walking conditions. These alterations may then impair performance and increase injury risk. Sensor-based technologies provide a practical pathway for detecting and monitoring these deficits in real-world environments, thereby supporting more precise return-to-activity decisions and advancing the application of measurement systems in human movement science.

The current laboratory-based findings identify spatial and temporal gait variables that may be useful targets for future sensor-based research. Step length, single-support, and double-support can be measured by several wearable platforms, but the present study did not test these systems outside the laboratory. Future studies should determine whether these gait alterations are detectable during daily activity or sport-specific movement and whether they predict recovery status or subsequent injury risk.

## Figures and Tables

**Table 1:** Mean time in seconds to completion for each trial per group.

Walking Condition	Concussed Group’s Adjusted Mean, 95% CI	Healthy Control Group’s Adjusted Mean, 95% CI
Single task w/no obstacle	19.2 s, 18.3–20.1	18.5 s, 17.6–19.5
Dual task w/no obstacle	22.8 s, 21.4–24.1	22.0 s, 20.6–23.4
Single task w/obstacles	21.9 s, 20.6–23.2	21.4 s, 20.0–22.7
Dual task w/obstacles	26.4 s, 25.0–27.8	25.5 s, 24.1–27.0

Note: CI denotes confidence interval.

**Table 2:** Average number of utterances, number of correct responses, and percentage of correct responses per group.

		Concussed Group	Healthy Control Group
Dual task with no obstacle	Number of utterances	4.3 ± 2.2	4.3 ± 2.2
	Number of correct responses	3.6 ± 2.6	5.0 + 2.7
	Percentage of correct responses	77.8 ± 30.8%	89.2 ± 16.0%
Dual task with obstacles	Number of utterances	4.8 ± 3.8	5.3 ± 2.6
	Number of correct responses	4.2 ± 2.9	4.8 ± 2.6
	Percentage of correct responses	83.6 ± 29.1%	89.5 ± 13.6%

Note: Values presented in mean ± SD.

**Table 3:** Average gait velocity and average step length across walking conditions per group.

	Walking Condition	Concussed Group’s Adjusted Mean, 95% CI	Healthy Control Group’s Adjusted Mean, 95% CI
Average Gait Velocity (cm/s)	Single task w/no obstacle	117.9, 112.3–123.5	123.0, 117.2–128.8
	Dual task w/no obstacle	103.1, 96.4–109.7	105.2, 98.3–112.1
	Single task w/obstacles	95.3, 87.9–102.6	100.9, 93.24–108.47
	Dual task w/obstacles	78.8, 72.3–85.3	84.7, 77.9–91.4
Average Step Length (cm)	Single task w/no obstacle	66.8, 64.6–69.1	70.1, 67.8–72.5
	Dual task w/no obstacle	62.5, 60.0–65.0	64.9, 62.3–67.5
	Single task w/obstacles	64.7, 59.2–70.1	73.5, 67.8–79.2
	Dual task w/obstacles	58.6, 53.6–63.5	69.3, 64.1–74.4

Note: CI denotes confidence interval.

**Table 4:** Mean percentage of single- and double-support across walking conditions and groups.

	Walking Condition	Concussed Group’s Adjusted Mean, 95% CI	Healthy Control Group’s Adjusted Mean, 95% CI
Single-Support Percentage	Single task w/no obstacle	35.9%, 35.3–36.5	35.9%, 35.2–36.5
	Dual task w/no obstacle	34.8%, 34.1–35.5	34.9%, 34.2–35.5
	Single task w/obstacles	39.5%, 38.3–40.3	40.5%, 39.7–41.3
	Dual task w/obstacles	39.2%, 38.5–39.8	39.9%, 39.3–40.6
Double-Support Percentage	Single task w/no obstacle	28.3%, 27.1–29.5	28.4%, 27.1–29.6
	Dual task w/no obstacle	30.4%, 29.1–31.7	30.6%, 29.2–31.9
	Single task w/obstacles	21.2%, 19.9–22.5	19.7%, 18.3–21.0
	Dual task w/obstacles	22.2%, 21.1–23.5	20.8%, 19.5–22.0

Note: CI denotes confidence interval.

## Data Availability

The raw data supporting the conclusions of this article will be made available by the authors upon request.

## Abbreviations

The following abbreviations are used in this manuscript:

BMI	Body mass index
MoCA	Montreal Cognitive Assessment
RMANCOVAs	Repeated-measures analyses of covariance
